# Identification of the Hydrogeochemical Processes in Groundwater Using Classic Integrated Geochemical Methods and Geostatistical Techniques, in Amol-Babol Plain, Iran

**DOI:** 10.1155/2014/419058

**Published:** 2014-01-12

**Authors:** Tahoora Sheikhy Narany, Mohammad Firuz Ramli, Ahmad Zaharin Aris, Wan Nor Azmin Sulaiman, Hafizan Juahir, Kazem Fakharian

**Affiliations:** ^1^Faculty of Environmental Studies, Universiti Putra Malaysia (UPM), Serdang, 43400 Selangor, Malaysia; ^2^Department of Civil and Environmental Engineering, Amirkabir University of Technology, Tehran 15875-4413, Iran

## Abstract

Hydrogeochemical investigations had been carried out at the Amol-Babol Plain in the north of Iran. Geochemical processes and factors controlling the groundwater chemistry are identified based on the combination of classic geochemical methods with geographic information system (GIS) and geostatistical techniques. The results of the ionic ratios and Gibbs plots show that water rock interaction mechanisms, followed by cation exchange, and dissolution of carbonate and silicate minerals have influenced the groundwater chemistry in the study area. The hydrogeochemical characteristics of groundwater show a shift from low mineralized Ca-HCO_3_, Ca-Na-HCO_3_, and Ca-Cl water types to high mineralized Na-Cl water type. Three classes, namely, C_1_, C_2_, and C_3,_ have been classified using cluster analysis. The spatial distribution maps of Na^+^/Cl^−^, Mg^2+^/Ca^2+^, and Cl^−^/HCO_3_^−^ ratios and electrical conductivity values indicate that the carbonate and weathering of silicate minerals played a significant role in the groundwater chemistry on the southern and western sides of the plain. However, salinization process had increased due to the influence of the evaporation-precipitation process towards the north-eastern side of the study area.

## 1. Introduction

Groundwater plays a major role in the water supply and ecology of arid and semiarid regions. The quality of groundwater is important in order to support life [1]. Groundwater is controlled by natural and anthropogenic factors, such as geological structure, composition of precipitation [2], geochemical process, the interaction between the groundwater and aquifer minerals [3], and human activities. The interaction of these factors result in various water types [4]. The groundwater chemistry depends on different hydrogeochemical processes that the groundwater undergoes over space and time.

In arid and semiarid areas, such as the Amol-Babol Plain in the north of Iran and to the south of the Caspian Sea, several processes evaporation, transpiration, seawater intrusion, cation exchange, dissociation and precipitation minerals, oxidation reduction, and biological processes could be involved in the groundwater chemical composition at the same time. Salinization is one of the principal water problem concerns that have become a major threat to the quality of the freshwater being suitable for human consumption. Land use activities, climate conditions, and the geological setting have a significant influence on the groundwater salinity [5]. Improper irrigation and drainage technique in arid and semiarid areas could also increase the risk of the progressive salinization of soil, because of the solute accumulation in irrigation water [2, 6]. Several studies have indicated the role of other processes such as high evaporation rate and limited discharge [7], excessive pumping of groundwater [8], fossil seawater [9], and seawater-freshwater mixing [10] which increases the salt concentration in the groundwater. The coastal area on the north side of the Amol-Babol Plain is under increasing human pressure from population growth and the increase in the development of agricultural activities. Groundwater resources along the shoreline could be threatened by the mixing mechanism of seawater intrusion into the freshwater, due to the over abstraction of groundwater [11, 12].

In as much as salinization on the Amol-Babol Plain may be caused by a combination of different processes; this research was undertaken to identify the source of salinity of groundwater and determine the hydrogeochemical process involved in the salinization of groundwater in the study area.

The weathering conditions, altitude, and geological structure are different in the southern part of the study area, where the plain is restricted by the Alborz Highlands. The hydrochemistry of the groundwater near the Highlands might be influenced through the dissolution of carbonate rocks, cation exchange, and the adsorption of dissolved ions as the dominant processes [1]. Hanshaw and Back [13] noted that carbonate aquifers constitute a dynamic geochemical system with the water-rock interaction due to the fast dissolution/precipitation kinetics of carbonate minerals.

Studies of the major ions have been used to identify the hydrochemical facies of the water. Several researchers have evaluated the groundwater chemistry and consider hydrogeochemical processes by developing geochemical modelling and adopting graphical methods for the interpretation of water quality indices [2, 14–16]. In recent years, multivariate statistical techniques coupled with PHREEQC software [17] and Geographical Information Systems (GIS) [18] have been applied to detect important information from the hydrogeochemical data in complex systems [4, 19–21]. This multidisciplinary approach will be useful to identify and locate different physiochemical processes in the groundwater in complex aquifers. The combination of hydrogeochemical tools and statistical analysis is applied to investigate the properties of groundwater among the sampling sites. The application of GIS provides a unified way to represent the physiochemical characteristic in a specific area, as well as the presentation of spatial distribution of groundwater hydrochemistry parameters in the thematic maps [22]. The GIS method based on Na^+^/Cl^−^, Mg^2+^/Ca^2+^, and Cl^−^/HCO_3_^−^ ionic ratio, and electrical conductivity (EC) is an attempt to understand the groundwater quality variations by the distribution maps [20]. Although GIS is an appropriate tool to map, query, and analyse the data [22], which could be effective for the authorities to manage the natural resource in a given certain area [23], not many studied have been conducted with this method in hydrochemical evaluation of groundwater.

The sustainable development of water resources in the arid and semiarid areas highly depends on the investigation of the hydrogeochemical evolution of groundwater. Nevertheless, limited research is available regarding the hydrochemical characteristics of the Amol-Babol Plain.

This study focuses on investigating the major hydrogeochemical aspects of groundwater chemistry on the Amol-Babol Plain as well as providing an overview of the spatial distribution of the groundwater ionic ratios by using GIS and interpolation techniques.

## 2. Materials and Methods

### 2.1. Study Area

**Figure 1:**
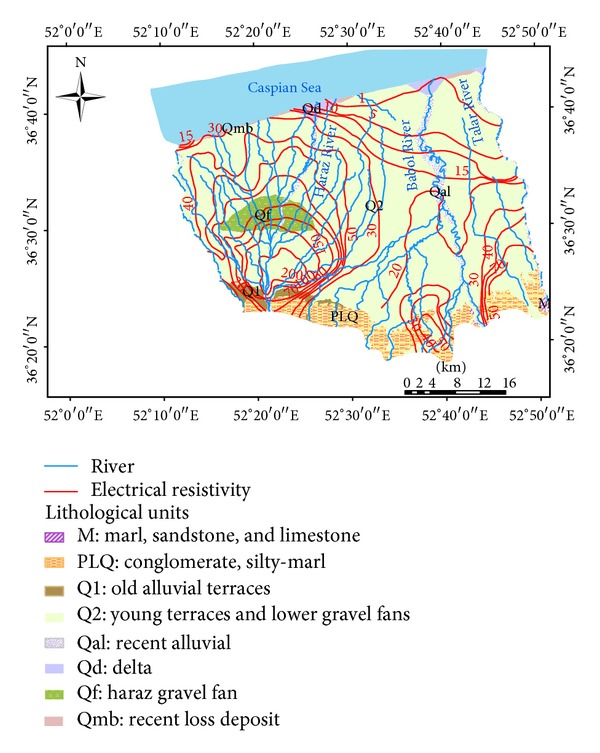
Geological map and electrical resistivity curve of the Amol-Babol Plain.

The study area is in the Amol-Babol Plain, which is located between longitudes 51°26′ and 52°55′E and latitudes 35°46′ and 36°43′N (Figure 1). It consists of a broad plain on the northern side of the Mazandaran Province which is bounded to the south by the Alborz Highlands and to the north by the Caspian Sea. The plain covers an area of 1822 km^2^. Topographically, the study area starts from the hillside of the Alborz Highland (above 970 m), which is covered by forest and irrigated lands in the valley. The plain has been formed by river deposits that developed alluvial fans, a flood plain, and marine deposits. The lowest elevation of about −25 m is observed in the coastal regions near the Caspian Sea.

The main annual temperature is about 17.9°C. The temperature decreases from the north of the area, which is close to the Caspian Sea to the south around the Alborz Highlands [24]. The annual precipitation is about 880 mm. Precipitation varies from 350 mm in the rainy season to 147 mm in dry season. The annual evaporation is about 750 mm in the middle of plain where the evaporation increases in proportion to the distance from the Caspian Sea [24].

Haraz, Babol, and Talar are the three largest rivers in the study area. The rivers begin from the Alborz Highlands and flow northward to the Caspian Sea. The Haraz River flows on the western side of the Amol-Babol Plain and passes through the City of Amol. The river is a major source of agricultural activity in the region. The Haraz River has a length of 185 km with an annual discharge of 940 mcm [24]. The Babol River passes through Babol City. The annual flow is 493 mcm. The Talar River on the eastern side of the study area flows to about 58 km from Shirgah to the Caspian Sea. The annual discharge rate is 311 mcm at Kiakola station.

Around 63% of the total water consumed is provided from the groundwater resources in the Amol-Babol Plain. More than 80% of the water resources for agricultural activities are supplied by the groundwater. Moreover, the groundwater is the main source for drinking in the study area.

### 2.2. Geology

The Amol-Babol Plain is part of the geological unit of the Georgian-Rasht Zone situated in the north of the Alborz great fault, which extends from Georgian to Lahijan [25]. The oldest geological formation of the study area dates back to the Permian age and includes Dorud, Ruteh, and Nessan formations, which mostly consist of a limestone layer and also thin layers of shale and marl [25]. These formations mostly outcrop in the southern parts of the study area near the Haraz road. When global de glaciation started in the Triassic age, sedimentation stopped in the Alborz area [25], and the sea level started to rise with the commencement of sedimentation during the Jurassic period. The bedrock of the area mostly belongs to the lower to middle Jurassic formation.

The lithology of the study area consists of conglomerates, sandstone, siltstone, shale, and coal seams. The late Jurassic is represented by the presence of limestone in the sediment layers. Cretaceous marl and clay cover top of the Jurassic limestone in the south and south-eastern side of the study area. During the Quaternary period, the process of deposition developed unconsolidated clay and sandy sediments with freshwater over an extensive part of the plain [25]. Alluvial fans have been formed by the weathering of rocks, transport and deposition of sediments by the rivers. During this process, Haraz homogenous and Talar heterogeneous alluvial fans had been extended to the western and eastern side of the Amol-Babol Plain [26]. The Caspian Sea is a remnant of the Paratethys Sea, which became an enclosed water body around 5.5 million years ago, because of tectonic uplift, a decrease in sea level and climate change [25]. Then within the last one million years, old sea water, which was trapped between the layers of sediment layers, was washed and discharged to the sea by the river and groundwater flows [25]. However, fossil saline water still remains in limited areas on the north eastern side of the plain, due to the weak discharge rate of the Talar River and the heterogeneous sediment layers of clay and sand in the study area [26].

### 2.3. Hydrogeology

Groundwater in the study area is represented by an unconfined aquifer and a confined aquifer. The unconfined aquifer extends to around 94% of the plain, except for a limited area on the north-eastern side. The thickness of alluvial deposits changes from less than 10 m for the coastal land to around 200 m for the Haraz alluvial fan in the west and south-western part of the plain. The groundwater tables decrease from the Highlands on the southern side to the coastal area (Figure 1).

**Figure 2:**
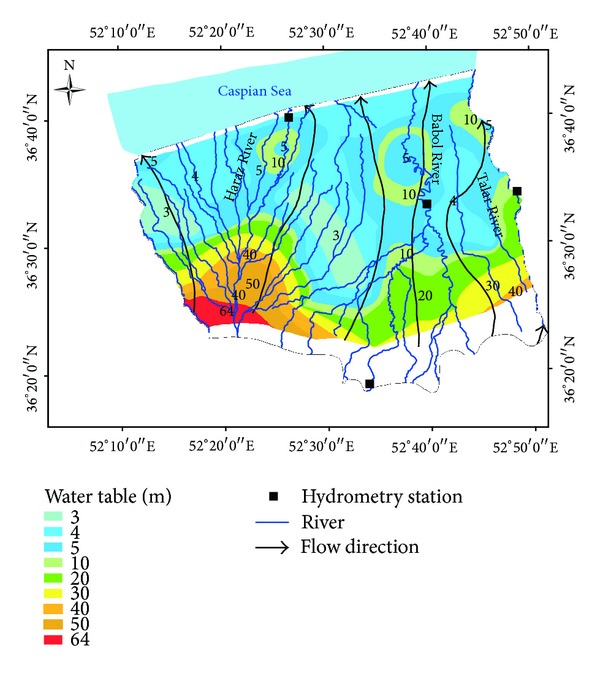
Schematic map of groundwater table in the study area.

The main direction of the groundwater flow is from the recharge zone (Alborz Mountain) in the south to the discharge area (Caspian Sea) in the north part of the plain (Figure 2). The hydraulic gradient varies from 10 per thousand in the southern area to 0.5 per thousand in the coastal plain of the Caspian Sea [26]. There are around 61,496 shallow and 6,634 deep wells on the Amol-Babol Plain. The water abstraction from the plain aquifer was about 390 million m^3^ for agricultural activities and around 44 million m^3^ for drinking purposes, during the 2008 to 2009 period [26]. The total abstraction of groundwater was 507 million m^3^ through the wells, springs, and quants, of which 342 million m^3^ (around 94%) was discharged from wells [26].

### 2.4. Groundwater Sampling and Analysis

A total of 306 groundwater samples were collected from 153 wells during the dry and wet seasons in 2009. After pumping out for 10–15 minutes to prevent nonrepresentative samples of stagnant or polluted water [27], the analyses were undertaken within 24 hours of the sampling exercise. The parameters, such as temperature, electrical conductivity (EC), pH, total dissolved solid (TDS), and dissolved oxygen (DO) were measured in the field immediately after sampling using a multiparameter WP600 series meter. The bottles were rinsed using the groundwater to be sampled. The samples were taken and stored in the acid-washed polyethylene bottles [28]. The bottles were rinsed using the groundwater to be sampled. Also, the samples were filtered using a 0.45 *μ*m, acetate cellulose filter on site [28, 29]. The collected samples were kept at 4°C and transported to the laboratory. The samples were analysed for sodium, potassium, calcium, magnesium, chloride, sulfate, bicarbonate, and carbonate based on the APHA [28] procedures in the laboratory. The Ca^2+^ and Mg^2+^ were determined titrimetrically using the standard EDTA method, and sodium and potassium by flame photometry. The anions and bicarbonate were determined by acid titration, while the chloride concentration was determined by AgNO_3_ titration and the sulfate and phosphate values by spectrophotometer. The quality of the analyses of the parameters to obtain a reliable dataset was controlled by sending the blank samples to the laboratory. The accuracy of the results was checked by calculating the ion balance errors, which was generally within ±5.

### 2.5. Statistical Analysis

Multivariate statistical analyses were applied to obtain significant information from hydro-chemical data sets in complex systems. Chemical variables were graphically interpreted using Piper, Schoeller, and Gibbs diagrams to show the groundwater facies for the Amol-Babol Plain. Multivariate statistical methods of descriptive statistics, Pearson correlation analysis, discriminant analysis (DA), and cluster analysis (CA) were performed as quantitative and independent methods for classification of groundwater samples and to correlate between the chemical parameters and groundwater samples. Multivariate statistical analysis utilizes normally distributed data [30]. The data for most chemical parameters are positively skewed. The data varied between 0 and +2. Only values within the range of −2 to +2 showed a normal distribution [31], F^−^, Na^+^, K^+^, NO_3_^−^, CO_3_^2−^, and pH were not found to be normally distributed (Table 2). The data were then log-transformed so that it will be normally distributed Prior to the multivariate analyses, all the parameters were standardized by subtracting the mean value and dividing by the standard deviation of parameters [16]. Discriminant analysis was applied to characterize and categorize the water quality into exclusive and exhaustive groups based on the relationship between dependent variable and independent variables [32]. Linear combinations of independent variables will discriminate the groups to minimize the misclassification error [33]. DA was applied on the raw data using forward stepwise methods to determine the most significant variables that highly influenced groundwater quality [34]. For the classification of variables based on the similarities within a class and heterogeneity between the classes cluster analysis was applied on the normalized dataset by means of the Ward method [35]. Hierarchical agglomerative cluster analysis (HACA) was mostly used where clusters were formed sequentially, by starting from the most similar pair of objects and forming higher clusters [36]. The dendrogram provides a visual summary of the clustering process, presenting a picture of classes and their proximity. In this study, the degree of association of relationship between two variables was summarized using Pearson's correlation analysis [30]. The values of correlation varied from +1 to −1. Where +1 indicates strongly positive correlation, −1 represents strongly negative correlation, and 0 means no linear correlation.

### 2.6. Identification of Salinization Zones

Geographical information system (GIS) and geostatistical techniques provide an integrated tool to investigate the spatial distribution of EC, Na^+^/Cl^−^, Mg^2+^/Ca^2+^, and Cl^−^/HCO_3_^−^ ionic ratios. The spatial distribution maps of each ratio, EC, and SAR were generated using the ordinary kriging method, which is one of the best interpolation methods in ARCGIS geostatistical extension [22]. Ordinary kriging is a method for linear optimum appropriate interpolation with a minimum square error of the un sampled location, based on the following [37]:

(1) $$\begin{array}{c} Z^{*}\left(x_{0}\right)=\overset{n}{\underset{i=1}{\sum }}n\lambda_{i}Z\left(x_{i}\right), \end{array}$$

where Z*(*x*_0_) is the estimated value at location *x*_0_, *n* is the number of the points, *Z*(*x*_*i*_) is the known value at location *x*_*i*_, and *λ*_*i*_ is the kriging weight. The basic geostatistical tool for modelling the spatial autocorrelation of a regionalized variable is the semivariogram, which measures the average degree of dissimilarity between the unsampled values and the nearest data values [38]. The experimental variogram's value for a separation distance of *h* is half the average squared difference between the value at *Z*(*x*_*i*_) and the value at *Z*(*x*_*i*_ + *h*) [39]:

(2) $$\begin{array}{c} \gamma \left(h\right)=\frac{1}{2n\left(h\right)}\overset{n(h)}{\underset{i=1}{\sum }}{\left[Z\left(x_{i}\right)-Z\left(x_{i}+h\right)\right]}^{2}, \end{array}$$

where *n*(*h*) is the number of data pairs within a given class of distance and direction. The best fitting variogram model has been chosen by calculating the experimental semivariogram and fitting alternative semivariogram, using cross validation. The nugget/sill ratio and the root-mean square error (RMSS) were applied to provide accurate prediction in semivariograms.

Based on Table 1, the exponential model was chosen as the fitted model for Ca/Mg, Na/Cl, Cl/HCO_3_, and EC. The nugget/sill ratio varied between 30 and 60% and the RMSS values ranged around one, implying that there is a relatively insignificant bias and a good estimation of prediction variability.

## 3. Results and Discussion

**Figure 3:**
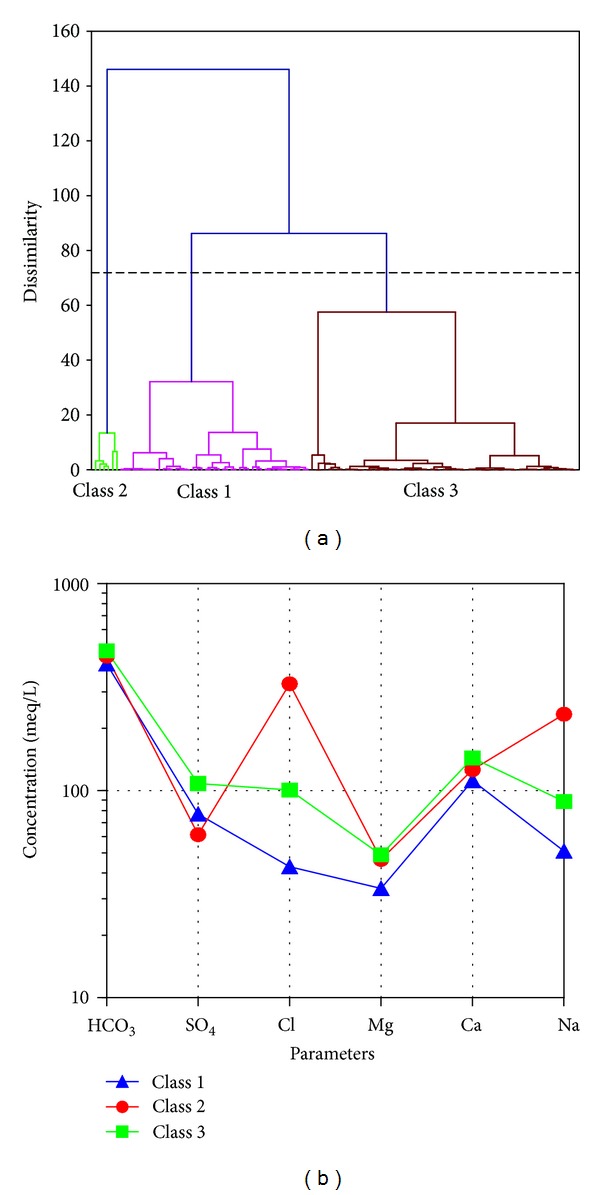
(a) Dendogram of the cluster analysis; (b) Schoeller diagram of the threea groundwater cluster.

The cluster analysis classified the groundwater samples into three general classes, namely, C_1_, C_2_, and C_3_, using cluster analysis based on the similarities among the chemical parameters involved in the groundwater quality on the plain (Figure 3(a)). The level of significance of the chemical parameters of sodium, magnesium, calcium, chloride, and electrical conductivity was determined by discriminant analysis according to spatial variation of the sampling wells in the study area.

The samples in classes C_1_ and C_3_ had a similar anionic composition that was dominated by HCO_3_^−^ with abundance orders of HCO_3_^−^ > Cl^−^ > SO_4_^2−^ (meq/L) (Figure 3(b)). The samples in clusters C_1_ and C_3_ had a cationic composition that was dominated by Ca^2+^ and Mg^2+^, respectively, with abundance orders of Ca^2+^≅Mg^2+^ > Na^+^ (meq/L). Therefore, the chemical compositions of these classes were characterized by the Ca-Mg-HCO_3_ water type. The samples in cluster one represented the freshwater type due to the mean concentrations of TDS of around 686 mg/L and EC about 939 *μ*S/cm, whilst the C_3_ water samples tended to brackish be water (average TDS = 1004 mg/L and average EC = 1372 *μ*S/cm). The samples in the second cluster showed a different ionic composition that was dominated by Na^+^, with abundance orders Na^+^ > Ca^2+^≅Mg^2+^ (meq/L) (Figure 3(b)) and the anionic composition was dominated by Cl^−^ > HCO_3_^−^ > SO_4_^2−^ (meq/L).

Thus, their chemical composition was characterized by the Na-Cl type, which could be supported by the mean TDS values being greater than 1549 mg/L and an average EC of around 2058 *μ*S/cm, which represents the brackish to saline water type.

### 3.1. Hydrogeochemical Facies

**Figure 4:**
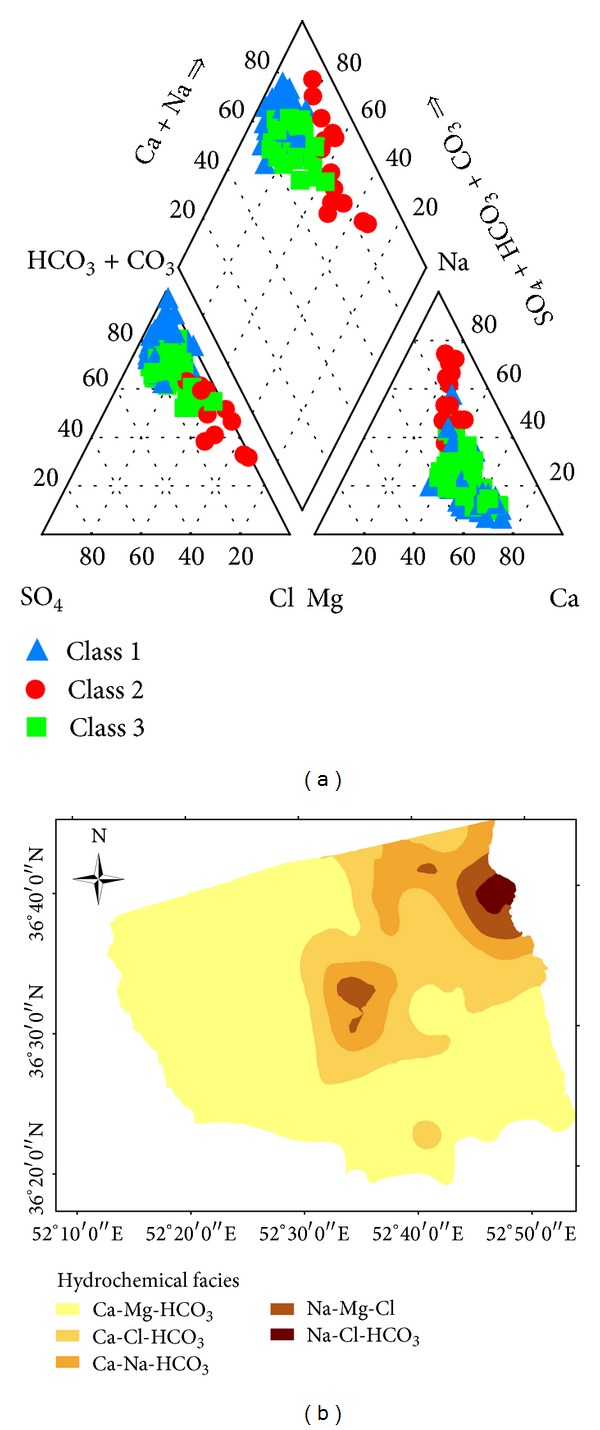
(a) Piper diagram presentation for groundwater constituents. (b) Spatial distribution of water type in the groundwater of the study area.

The Schoeller diagram showed that Ca^2+^ and HCO_3_^−^ are the dominant ions in most parts of the study area (Figure 3(b)). The majority of groundwater samples showed Ca-Mg-HCO_3_, mixed Ca-Cl and Ca-Na-HCO_3_, and NaCl types. About, 71% of the groundwater samples were characterized as Ca-HCO_3_ type, due to the carbonate dissolution process and wide contact with limestone. The groundwater type changed to Ca-Cl, Ca-Na-HCO_3_, and Na-Cl as it moved from the west and south side towards the eastern and north-eastern sides of the plain (Figure 4(b)). The Ca-HCO_3_ water, which extended in the Haraz alluvial fan and the southern side of the Alborz Highlands, represented permanent hardness of the groundwater. Towards the center of the plain, the groundwater type changed to Ca-Cl and Ca-Na-HCO_3_, which indicated the slightly saline water. To the east and north-eastern sides of the plain Na^+^ was the dominant cation, while Cl^−^ was the dominant anion. The groundwater samples were classified as Na-Cl type, which represented the saline water type. The spatial distribution map showed that the groundwater type gradually changed from freshwater on the south and western sides to saline water on the north-eastern side of the plain (Figure 4(b)).

### 3.2. Correlation of Major Ions

In general, the groundwater was found to be slightly acidic with pH values varying from minimum 6.41 to a maximum of 7.67 especially on the southern side (class 1) where it was covered by carbonate rock formations. Naturally, rainwater is slightly acidic [40], due to the reaction with carbon dioxide in the atmosphere, thereby making the rainwater slightly acidic, according to (1) (Table 1):

(3) $$\begin{array}{c} {\text{H}}_{\text{2}}\text{O}+{\text{CO}}_{\text{2}}⟶{\text{H}}_{\text{2}}{\text{CO}}_{\text{3}}\;\left(\text{Carbonate}\;\;\text{Acid}\right) \end{array}$$

The carbonate acid in the water could be breaks down based on (4), producing (HCO_3_) and H^+^:

(4) $$\begin{array}{c} {\text{H}}_{\text{2}}{\text{CO}}_{\text{3}}⟷\text{H}{\text{C}{\text{O}}_{3}}^{-}+{\text{H}}^{+}\\ {\text{HCO}}_{\text{3}}⟷{\text{H}}^{\text{+}}+{\text{C}{\text{O}}_{3}}^{2-} \end{array}$$

The alkalinity of water is the measure of its capacity for neutralization [41], which is represented by the bicarbonate (HCO_3_^−^). HCO_3_^−^ was the dominant anion, which varied from a minimum of 182.5 mg/L to a maximum of 1065 mg/L in the study area. The concentration of HCO_3_^−^ showed spatial variation, due to the existence of carbonate rock in the recharge area.

Significant differences were observed in the total dissolved solids, where the TDS values vary from a minimum of 414 mg/L (from class 1) to a maximum of 2441 mg/L (from class 2). The large variations of the EC and TDS in the groundwater were dependent on the geochemical processes and the anthropogenic activities, such as the application of fertilizers and seawater intrusion in the study area.

The high values of TDS concentration represented a high concentration of dissolved ions in the groundwater samples, which strongly correlated with the EC values (*r* = 0.985; *P* < 0.05), Cl^−^ concentration (*r* = 0.719; *P* < 0.05), and Na values (*r* = 0.853; *P* < 0.05) (Table 3). The strong correlation between TDS, EC, Na, and Cl^−^ showed that these ions could be derived from the same source. Moreover, there was a significant correlation (*r* = 0.737; *P* < 0.05) between Na and Cl, which indicated that groundwater salinity in the plain may have originated from three sources seawater intrusion, evaporated deposits, and fossil saline water entrapped in the sediments. From the analysis of the major ions, it was observed that Ca^2+^ and Mg^2+^ were the dominant cations, with the exception of samples belonging to class two, which were characterized by a high concentration of Na^+^ and K^+^. The calcium concentration was as high as 175 mg/L and as low as 20 mg/L.

High calcium concentration might have originated from calcite and dolomite weathering or silicate rock dissolution. Ca^2+^ and Mg^2+^ constitute the possible sources of hardness, which were common in the limestone areas.

The groundwater hardness showed a strong correlation with Ca^2+^ (*r* = 0.746) and Mg^2+^ (*r* = 0.775), which reflected that they originated from the same sources. The concentration of potassium in the groundwater samples varied from 0.58 to 82.9 mg/L on the plain. Although, high potassium concentrations have been derived from anthropogenic sources, such as potash feldspar in the agricultural lands, which were mostly observed in the central part of the plain, the weak correlations between K^+^ and other major ions suggested that potassium mostly originated from k-feldspar or k-bearing minerals (Table 3) [2].

### 3.3. Ionic Ratio

**Figure 5:**
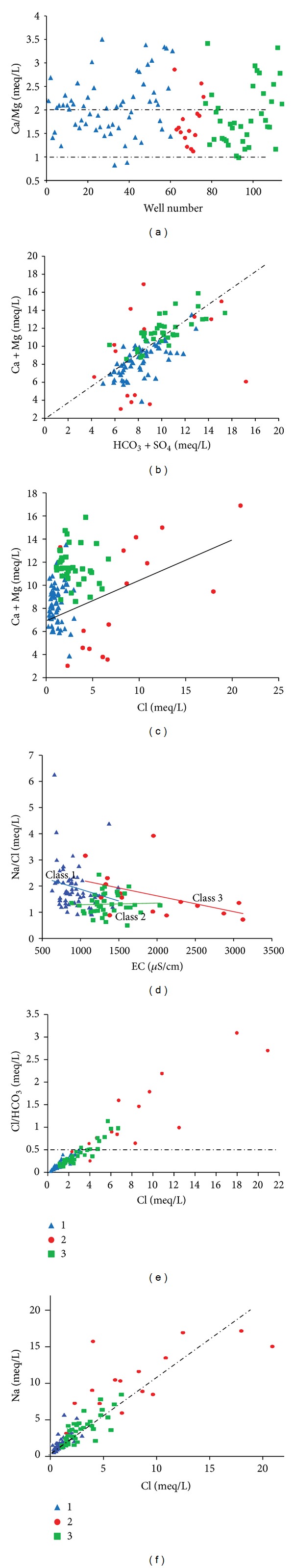
Distribution of ionic ratios for major groundwater ions from the study area.

**Figure 6:**
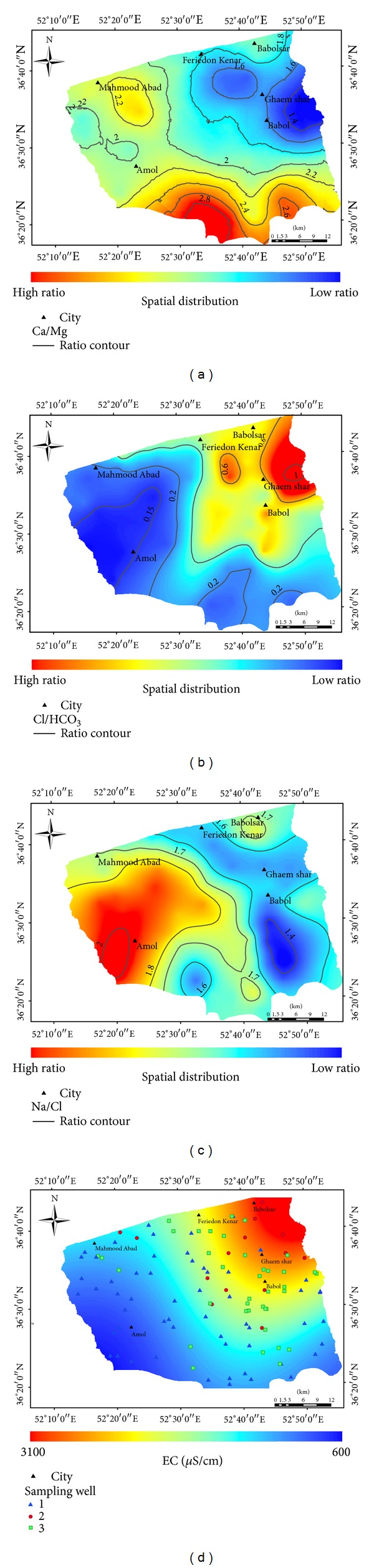
Spatial distribution of (a) Ca/Mg ratio, (b) Cl/HCO_3_ ratio, (c) Na/Cl ratio, and (d) electrical conductivity, of groundwater in Amol-Babol Plain.

Calcium and magnesium were the dominant cations and bicarbonate was the dominant anion in wide areas of the western, eastern, and central sides of the plain. The abundance of Ca^2+^ and Mg^2+^ in the groundwater could be related to the presence of carbonate rock in the basin, while weathering of carbonate and silicates may contribute Ca^2+^ and Mg^2+^ in the groundwater. The dissolution of calcite and dolomite can be shown by the groundwater's Ca^2+^/Mg^2+^ molar ratio. A Ca^2+^/Mg^2+^ molar ratio that is equal to one indicates dissolution of dolomite rocks [42], while a greater ratio may represent a more dominant calcite contribution from the rocks. A Ca^2+^/Mg^2+^ ratio, greater than 2, may represent the dissolution of silicate minerals into the groundwater [43]. Whilst 59% of the groundwater samples had A Ca^2+^/Mg^2+^ ratio between 1 and 2, which indicated that the dissolution of calcite 38% of the samples had a higher ratio than 2, which showed the effect of silicate minerals that contribute calcium and magnesium to the groundwater [42]. Only a few samples (around 2.6%) were indicative of the dissolution of dolomite with Ca^2+^/Mg^2+^ ratio <1 (Figure 5(a)). The spatial pattern in the ratio of calcium and magnesium showed the variation from the south to the central and eastern parts of the plain (Figure 6(a)). The ratio was the highest towards the Alborz Highlands on the southern side, due to the increase in calcium concentration through the weathering of silicate and carbonate rocks in the recharge area. The ratio decreased with the distance from the weathering zones towards the discharge area in the Caspian Sea (Figure 6(a)). The dissolution of carbonate minerals could be represented in the following reactions ((5) to (8)) in natural systems [44]:

(5) $$\begin{array}{c} \text{Ca}{\text{CO}}_{3}\;\left(\text{Calcite}\right)+{\text{H}}_{2}{\text{CO}}_{3}⟶{\text{Ca}}^{2+}+2\text{H}{\text{C}{\text{O}}_{3}}^{-} \end{array}$$

(6) CaMg(CO3)2(Dolomite)+H2CO3  ⟶Ca2++Mg2++4HCO3−

(7) $$\begin{array}{c} {\text{CaCO}}_{3}+{\text{H}}_{2}{\text{SO}}_{4}⟶{\text{Ca}}^{2+}+{\text{S}{\text{O}}_{4}}^{-}+{\text{H}}_{2}{\text{CO}}_{3} \end{array}$$

(8) CaMg(CO3)2+2H2SO4  ⟶Ca2++Mg2++2SO4−+2H2CO3

Dissolution is a simple and common weathering reaction in carbonate rocks [45]. It is specified by the 1 : 2 ratio of Ca^2+^/HCO_3_^−^, and 1 : 1 equivalent ratio of Ca^2+^ + Mg^2+^/HCO_3_^−^, in the groundwater. The mean value of Ca^2+^/HCO_3_^−^ ratio was 0.79, which was near the 1 : 2 ratios and represented the significance influence of dissolution in the carbonate rocks. The Ca^2+^ + Mg^2+^/HCO_3_^−^ mean ratio in the groundwater was 1.38 that is also near to 1 : 1 equivalence ratio, representing that around 61% of the bicarbonate is related to calcium and magnesium. The lower value of Ca^2+^ + Mg^2+^/HCO_3_^−^ was observed in about 5% of the samples that indicative of other sources of HCO_3_ such as silicate weathering in the study area. Around 34% of samples showed the higher ratio of Ca^2+^ + Mg^2+^ to HCO_3_ that represents that the excess of Ca^2+^ and Mg^2+^ has been balanced by Cl^−^ and SO_4_^2−^. The plot of Ca^2+^ + Mg^2+^ versus HCO_3_^−^ + SO_4_^2−^ will be near to 1 : 1 line if Ca^2+^, Mg^2+^, SO_4_^2−^, and HCO_3_^−^ are derived from the dissolution of calcite, dolomite, and gypsum. If ion exchange is the dominant process, the data points tend to shift to the right due to excess of SO_4_^2−^ + HCO_3_^−^. The ion exchange reaction may be explained as follows:

(9) $$\begin{array}{c} \frac{1}{2}\text{Ca}-{\text{Clay}}_{2}+{\text{Na}}^{+}⟶\frac{1}{2}{\text{Ca}}^{2+}+\text{Na}-\text{Clay} \end{array}$$

If the points were above the median line, a reverse ion exchange was the active reaction for the excess of Ca^2+^ + Mg^2+^ over SO_4_^2−^ + HCO_3_^−^, which could be represented by the following reaction:

(10) $$\begin{array}{c} \text{Na}-\text{Clay}+\frac{1}{2}{\text{Ca}}^{2+}⟶{\text{Na}}^{+}+\frac{1}{2}\text{Ca}-{\text{Clay}}_{2} \end{array}$$

The average ratio of Ca^2+^ + Mg^2+^/HCO_3_^−^ + SO_4_^2−^ was 1.09. Based on the plot, the majority (73.6%) of samples were scattered close to the 1 : 1 line, indicating that an excess of Ca^2+^ and Mg^2+^ in the groundwater may be due to the dissolution of calcite, dolomite, and gypsum.

Moreover, around 14.7% of the samples fall above the median line, indicating that reverse ion exchange tended to be the dominant reaction over ion exchange, which was responsible for the higher HCO_3_^−^ and SO_4_^2−^ concentration in the groundwater (Figure 5(b)).

The plot of Ca^2+^ + Mg^2+^ versus Cl^−^ and Na^+^/Cl^−^ versus Cl^−^ clearly indicated that the salinity increased with a decrease in Na^+^/Cl^−^ and an increase in Ca^2+^ + Mg^2+^, which may be due to reverse ion exchange in the clay/weathered layer (Figures 5(c) and 5(d)).

The aquifer matrix may adsorb dissolved sodium in exchange for bound Ca^2+^ and Mg^2+^. Na^+^ was a dominant cation and Cl^−^ was a dominant anion on the east and north eastern side of the plain, which were mostly classified as the second cluster group. The high concentration of sodium and chloride in the groundwater could be related to the weathering of silicate rocks, the evapotranspiration process and/or seawater intrusion.

The Cl^−^/HCO_3_^−^ ratio may show the influence of salinization due to the seawater mixing to the groundwater. The ratio of Cl^−^/HCO_3_^−^ versus Cl ranged from 0.02 to 3.09 and showed a strong positive linear relation to the Cl^−^ concentration (*r* = 0.89, *P* < 0.01) (Figure 5(e)). About 80.7% of groundwater samples showed Cl^−^/HCO_3_^−^ ratios lower than 0.5, which means the groundwater was unaffected or freshwater.

The remaining samples fell between the ratios of 0.5–6.6 Cl/HCO_3,_ which showed that the water was slightly or moderately affected by salinization. The spatial distribution map of Cl^−^/HCO_3_^−^ ratio indicated that the unaffected water mostly covered the south and western side of the plain. However, salinization influenced the groundwater towards the east and north-eastern side of the study area, which was suspected to be affected by fossil saline water (Figure 6(b)).

The relationship between Na^+^-Cl^−^ has been used to identify the process that controls the salinity and saline intrusion in arid and semiarid areas [2, 46]. The origin of sodium concentration can be from different processes in the groundwater. The average molar ratio of Na^+^/Cl^−^ was 1.70 in the study area, which indicated higher Na^+^ values than the Cl^−^ (Figure 5(f)). The majority of the samples showed a Na^+^/Cl^−^ ratio equal to or greater than 1 that may represents sodium, which had been released from the silicate weathering process. Silicate weathering is the reaction of the feldspar minerals with the carbonate acid in the water, which is specified by bicarbonate as a dominant anion in the groundwater [47], similar to the Amol-Babol Plain (Figure 3(b)).

Halite is a dominant source of Na^+^ and Cl^−^ in the groundwater; the molar ratio varies spatially as a result of the cation exchange [48]. The availability of free halite for dissolution in the soil zone may increase in the arid and semiarid regions with low annual precipitation of less than <600 mm [49]. The ratio of Na^+^/Cl^−^ being equal to one indicated that halite dissolution could be responsible for the sodium concentration in the water samples. Based on the spatial distribution map of the Na^+^/Cl^−^ ratio, the majority of the study area was covered with a ratio greater than 1.5, which indicated the role of silicate weathering as the source of sodium in the study area (Figure 6(c)). Limited zones on the eastern side near the Babol and Ghaemshahr City represent a ratio near to one. Groundwater salinity may also relate to the formation of salt layers by leaching from the soil surface during evaporation in a semiarid climate. In the plot of Na^+^/Cl^−^ versus EC, the Na^+^/Cl^−^ showed a decreasing trend with increasing EC along with a higher a Na^+^/Cl^−^ ratio in class 1, which indicated that the Na^+^ originated from the silicate weathering process (Figure 5(d)).

Moreover, most of the samples belonging to classes 2 and 3 were plotted parallel to the electrical conductivity axis (Figure 5(d)) thereby indicating the role of evaporation and evapotranspiration to increase sodium concentration.

The electrical conductivity increased gradually from the west and the south to the north-eastern side of the plain (Figure 6(d)). The suspected area for the evaporation process, which was mostly covered with classes 2 and 3, showed the greater values of electrical conductivity. The silicate weathering process was found on the south and western side of the plain, which were monitored by the sampling well from class 1 (Figure 6(d)).

### 3.4. Gibbs Plot

**Figure 7:**
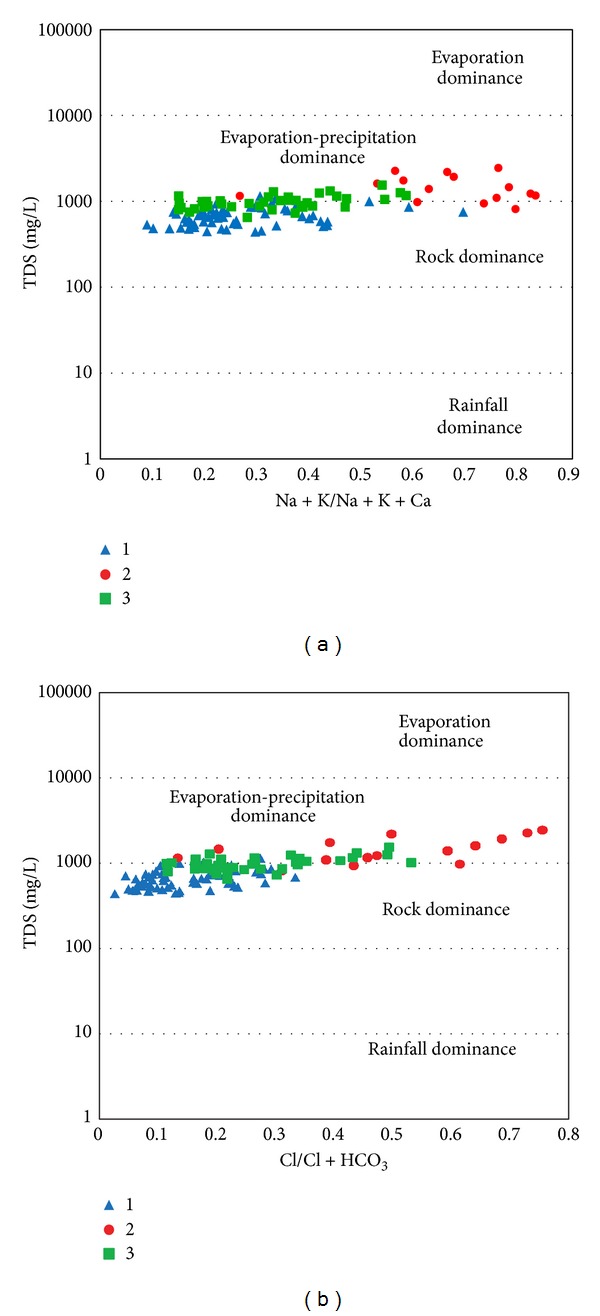
Gibbs plots explain groundwater chemistry and geochemical process in the study area.

Several factors control groundwater chemistry, which can be related to the physical situation of the aquifer, bedrock mineralogy and weather condition. Gibbs (1970) suggested TDS versus Na^+^/Na^+^ + Ca^2+^ for cations and TDS versus Cl^−^/(Cl^−^ + HCO_3_^−^) for anions to illustrate the natural mechanism controlling groundwater chemistry, including the rainfall dominance, rock weathering dominance, and evaporation and participation dominance. Based on the Gibbs diagram, the entire samples plotted fell into group one and 60% of the samples of group three may have been influenced by rock weathering reaction (Figure 7). The chemistry of all the samples belonging to class two and around 30% of samples from class three were controlled by the evaporation-precipitation dominance field (Figure 7). It seems that the ion chemistry of fresh groundwater (class 1 and most of class 3) generally related to carbonate and silicate weathering process on the south and west side of the study area. However, evaporation was the secondary factor that mostly control the groundwater chemistry in the north-eastern part where the class 2 sampling wells were situated.

### 3.5. Saturation Index

The interaction between the groundwater and rocks controls the geochemistry of the groundwater. The mineral equilibrium calculation can predict the thermodynamic control on the composition of the groundwater that has equilibrated with various minerals [50]. The saturation index was applied to predict the reactive mineralogy of the subsurface from the groundwater sample data without collecting the samples of the solid phase and analysing the mineralogy [50]. The saturation index (SI) was calculated using the computer geochemical program PHREEQC for groundwater samples [51], which can be defined as

(11) $$\begin{array}{c} \text{SI}=\log\left(\frac{\text{IAP}}{K}\right), \end{array}$$

where IAP is the Ion Activity product and *K* is the equilibrium constant. Equilibrium is indicated when SI = 0; the groundwater is supersaturated when SI > 0, which shows that precipitation is needed to achieve equilibrium. If SI < 0, the groundwater is under saturated; this indicates that dissolution is required to reach equilibrium.

The first and third groups showed slightly different behaviour from class 2 with respect to their carbonate minerals (Table 4). In classes 1 and 3, groundwater was supersaturated compared to the calcite and less saturated when compared to the dolomite and aragonite. In class 2, groundwater was supersaturated with calcite, dolomite, and aragonite.

The super saturation of groundwater with these carbonate minerals suggested that these carbonate minerals were the main components in the host rock. However, the lower saturation of these minerals has influenced the chemical composition of the groundwater. The evaporated minerals including, halite, gypsum, and anhydrite were under saturated in all groups, indicating that the soluble component Na^+^, Cl^−^, Ca^2+^, and SO_4_^2−^ concentration was not limited by mineral equilibrium [4].

## 4. Conclusions

The integration of statistical approaches and geochemical methods were applied to investigate the regional factors and processes governing the chemical composition of groundwater on the Amol-Babol Plain. In general, the dominant hydrogeochemical facies of groundwater were the Ca-HCO_3_ type, which covers large parts of the western, southern, and central sides of the plain, and changes to the Ca-Cl and Ca-Na-HCO_3_ types on the centre and the east side and Na-Cl type on the north-eastern side of the study area. The groundwater chemistry was mainly controlled by the weathering of minerals. Water rock interaction, including dissolution of carbonate mineral and silicate weathering, was the major hydrogeochemical processes that affect the groundwater hardness on the Amol-Babol Plain. The excessive sodium concentration could be due to the weathering of silicate rocks on the south and western sides of the plain, whereas evaporation was the dominant process in addition to the chloride on the east and north-eastern sides of the study area. Based on the hierarchical cluster analysis of sampling wells, samples of the first and third classes, which were distributed on the west, south, and central sides, represent the fresh-water type. The Gibbs plot indicated that the hydrochemistry of these groups were mainly influenced by the rock dominance, including carbonate and silicate rocks weathering. The groundwater type of second class gradually changed to saline water towards the north-eastern side of the plain. The Gibbs diagram clearly displayed the role of the evaporation process in the groundwater chemistry of the second group. The groundwater samples were saturated with respect to calcite and dolomite in all three groups, and were under saturated with respect to halite and gypsum minerals. Special management could be suggested for the salinity control in the areas with saline water, which were specified by spatial distribution maps in the ArcGIS environment.

## Figures and Tables

**Table 1:** Variographic parameters of the groundwater chemical composition.

Ratio	Model	Nugget (*C*_0_)	Sill (*C*_0_ + *C*)	(*C*_0_/*C*_0_ + *C*)∗100 Ratio	RMSS
Ca/Mg	Exponential	0.043	0.1214	33.9%	0.921
Na/Cl	Exponential	0.125	0.1875	60.1%	1.058
Cl/HCO_3_	Exponential	0.396	0.9891	40.0%	1.068
EC (*μ*S/cm)	Exponential	0.047	0.1458	32.3%	1.166

**Table 2:** Descriptive statistical analysis for the 153 groundwater samples of Amol-Babol Plain.

Parameters	Class 1					Class 2					Class 3				
	Min.	Max.	Mean	SD.	Skewness	Min.	Max.	Mean	SD.	Skewness	Min.	Max.	Mean	SD.	Skewness
Temperature	17.8	23.75	19.93	1.172	0.81	19.05	21.65	20.33	0.9	−0.08	19	23.45	20.2	1.02	1.37
pH	6.52	7.44	6.92	0.2	0.33	6.47	7.52	6.98	0.3	0.03	6.55	7.67	6.82	0.21	2.26
EC	628.3	1951	934.6	1.172	1.65	1268	3120	2026.2	695.2	0.41	881.8	2039	1337.8	236.9	0.71
TDS	440.5	1461	689.4	194	1.38	943.7	2441	1542.6	525.7	0.45	649.5	1544	989.3	185.5	0.74
DO	1.26	6.67	3.35	1.41	0.45	1.13	4.29	2.37	0.96	1.05	1.17	5.96	3.02	1.29	0.55
CO_3_^2−^	25	150	82.41	29.62	0.53	50	120	82.96	18.44	0.36	30	425	85.07	63.4	4.07
HCO_3_^−^	222.5	960	409	119	1.84	257.5	785	446	171.9	1.03	310	765	472.9	106.9	1.00
SO_4_^2−^	0.5	142.5	77	36.25	−0.38	0.5	170	61.26	54.89	0.73	57.5	165	108.2	33.48	0.08
F^−^	0.1	2.57	0.59	0.34	3.18	0.11	1.06	0.56	0.31	0.09	0.15	1.21	0.64	0.25	0.13
Ca^2+^	48.5	185	122.7	26.38	0.09	43.03	234.7	120.1	59.54	0.11	39.02	245.8	143.8	36.1	−0.35
Na^+^	13.13	362	51.02	46.66	5.00	72.24	394.4	233.9	105.8	0.05	27.3	207.9	88.66	48.11	0.84
Mg^2+^	17.6	66.11	33.69	10.03	1.01	16.93	78.4	46.5	20.6	−0.09	12.97	81.28	48.9	14.4	0.07
K^+^	0.58	82.91	5.5	10.85	0.19	0.8	19.42	6.68	5.38	0.88	0.82	35.81	6.08	6.89	2.58
Cl^−^	9	142.5	42.9	27.43	1.32	54	742	328.1	189.8	0.92	39	237.5	100.7	50.09	0.99
NO_3_^−^	0.061	83.26	7.96	14.81	3.80	0.06	15.2	5.25	5.09	0.73	0.02	24.2	5.06	3.12	1.71

**Table 3:** Correlation coefficient matrix of groundwater samples of Amol-Babol Plain.

Variables	Tem	pH	COND	TDS	DO	K^+^	Mg^2+^	Ca^2+^	CO_3_^2−^	HCO_3_^−^	SO_4_^2−^	Cl^−^	Na^+^	F^−^	TH	NO_3_^−^
Tem		0.432	0.045	0.000	0.027	0.260	0.836	0.196	0.745	0.672	0.159	0.395	0.018	0.022	0.334	0.709
pH	0.064		0.071	0.134	0.047	0.193	0.001	0.000	0.508	0.002	0.000	0.178	0.031	0.186	0.000	0.000
COND	0.162	−0.146		0.000	0.001	0.078	0.000	0.032	0.208	0.001	0.050	0.000	0.000	0.053	0.000	0.287
TDS	**0.306**	−0.122	**0.985**		0.000	0.074	0.000	0.093	0.226	0.002	0.037	0.000	0.000	0.026	0.000	0.355
DO	−0.179	0.161	−0.263	−0.287		0.308	0.031	0.038	0.181	0.915	0.016	0.016	0.005	0.000	0.901	0.010
K^+^	0.092	−0.106	0.143	0.145	−0.083		0.583	0.705	0.014	0.000	0.382	0.246	0.564	0.350	0.898	0.028
Mg^2+^	−0.017	−0.269	**0.373**	0.351	−0.174	0.045		0.052	0.142	0.070	0.000	0.015	0.035	0.020	0.000	0.179
Ca^2+^	−0.105	−0.399	0.174	0.136	0.168	−0.031	0.158		0.476	0.124	0.001	0.274	0.164	0.747	0.000	0.000
CO_3_^2−^	−0.027	0.054	0.102	0.099	−0.109	−0.198	0.119	−0.058		0.001	0.802	0.151	0.087	0.537	0.595	0.574
HCO_3_^−^	0.035	−0.254	0.267	0.253	−0.009	0.328	0.147	0.125	−0.275		0.069	0.853	0.191	0.000	0.027	0.002
SO_4_^2−^	0.114	−0.296	0.158	0.169	−0.195	0.071	0.365	0.276	−0.020	0.147		0.763	0.320	0.000	0.000	0.901
Cl^−^	0.069	0.109	**0.745**	**0.719**	−0.195	0.094	0.197	0.089	0.117	0.015	0.025		0.000	0.954	0.019	0.497
Na^+^	0.191	0.175	**0.856**	**0.853**	−0.226	0.047	0.171	−0.113	0.139	0.106	−0.081	**0.737**		0.118	0.598	0.178
F^−^	0.184	−0.108	0.157	0.180	−0.335	0.076	0.188	−0.026	0.050	0.311	**0.447**	0.005	0.127		0.177	0.328
TH	−0.079	**−0.436**	0.363	0.324	−0.010	0.010	**0.775**	**0.746**	0.043	0.179	**0.423**	0.189	0.043	0.110		0.000
NO_3_^−^	−0.030	−0.282	0.087	0.075	0.208	0.177	0.109	0.341	−0.046	0.244	0.010	−0.055	−0.110	−0.080	0.292	

Values in bold are different from 0 with a significance level alpha = 0.05.

**Table 4:** Statistical summary of saturation indexes of minerals in groundwater using PHREEQC.

	Anhydrite	Aragonite	Calcite	Dolomite	Gypsum	Halite
Class 1						
Min	−4.239	−0.437	−0.289	−1.005	−4.014	−8.348
Max	−1.500	0.577	0.725	1.034	−1.268	−6.560
Mean	−1.996	−0.076	0.072	−0.096	−1.759	−7.437
Cv	0.215	0.030	0.030	0.133	0.216	0.192
SD	0.464	0.174	0.174	0.365	0.464	0.439

Class 2						
Min	−4.170	−0.277	−0.129	−0.403	−3.931	−7.021
Max	−1.488	0.457	0.605	0.821	−1.254	−5.228
Mean	−2.363	0.080	0.227	0.303	−2.127	−5.882
Cv	0.589	0.036	0.036	0.135	0.587	0.216
SD	0.767	0.189	0.189	0.367	0.766	0.465

Class 3						
Min	−2.329	−0.497	−0.350	−0.968	−2.093	−7.560
Max	−1.471	0.290	0.438	0.539	−1.235	−5.953
Mean	−1.723	−0.075	0.072	−0.042	−1.486	−6.780
Cv	0.031	0.023	0.023	0.100	0.031	0.167
SD	0.177	0.151	0.151	0.316	0.177	0.409
